# Use of a Tissue Engineered Human Skin Model to Investigate the Effects of Wounding and of an Anti-Inflammatory on Melanoma Cell Invasion

**DOI:** 10.1371/journal.pone.0156931

**Published:** 2016-06-07

**Authors:** Claudia Mirian de Godoy Marques, Sheila MacNeil

**Affiliations:** 1 Department of Health Sciences, Santa Catarina State University, Florianópolis, State of Santa Catarina, Brazil; 2 Department of Materials Science and Engineering, Kroto Research Institute, University of Sheffield, Sheffield, South Yorkshire, United Kingdom; King's College London, UNITED KINGDOM

## Abstract

An increasing number of studies suggest inflammation stimulates tumour invasion. In melanoma, despite recent advances in targeted therapy and immunomodulatory therapies, this cancer remains difficult to treat. Our previous studies show melanoma cells interact with skin cells in their invasion into tissue engineered skin and suggest inflammation stimulates invasion. The aim of this study was to investigate the use of an anti-inflammatory on melanoma invasion. To do this we developed a wounded and inflamed in vitro 3D melanoma model in which to investigate the use of an anti-inflammatory on melanoma invasion. The tissue engineered skin model was based on human de-epidermised acellular dermis to which keratinocytes, fibroblasts and three different melanoma cell lines were added in various combinations. A simple incisional wound was made in the model and TNF-α and fibrin were added to simulate conditions of inflammation. Topical ibuprofen in a hydrogel was added and the extent of melanoma invasion into the dermis was assessed under the various conditions. The results showed that penetration of two of the cell lines (HBL and A375SM) into the tissue engineered skin was exacerbated by wounding and ibuprofen significantly decreased invasion of A375SM cells and slightly reduced invasion of HBL cells. A third cell line, C8161, was aggressively invasive under all conditions to an extent that was not influenced by wounding, TNF-α or the addition of ibuprofen. In summary, the results for one these cell lines (and a trend for a second cell line) support the hypothesis that a wound environment is conducive to melanoma invasion but the local addition of an anti-inflammatory drug such as ibuprofen may attenuate invasion.

## Introduction

Melanoma affects millions of people worldwide [1–4] and its incidence is increasing every year. While surgical treatment is successful for thin and superficial melanoma which are detected at an early stage, for melanoma thicker than 1mm at presentation the prognosis remains poor due to the aggressive invasion of these transformed melanocytes. The treatments available are essentially the surgical removal of the primary tumour and melanoma in the lymph nodes followed by chemotherapy. Historically metastatic melanoma has been one of the most difficult cancers to treat showing little response to conventional chemotherapy drugs. However recent years have seen improvements in survival time with drugs targeted to BRAF and MEK gene mutations in these cancers and with the use of newer immunomodulatory therapies targeted to checkpoint inhibitors.

Thus vemurafenib and trametinib respectively are used to target melanoma cells with BRAF and MEK gene mutations [5]. Post lymph-node dissection and therapy with BRAF and MEK inhibitors increased survival is reported. For example, vemurafenib has been found to be safe in patients with BRAF (V600) mutated metastatic melanoma [6], and combined therapy of drabrafenib and trametinib significantly improved overall survival in comparison to vemurafenib monotherapy alone [7]. Another combined therapy of vemurafenib and cobimetinib in patients with advanced BRAF (V600)-mutant melanomas has also been reported to be promising [8]. While immunotherapeutic drugs such as interferon and anti-CTLA4 antibodies remain under clinical investigation [9], the newer immunotherapies ipilimumab given with MAPK-targeted vemurafenib, dabrafenib and trametinib have demonstrated long term improvement in patient outcome, a benefit not afforded by traditional therapeutics [10].

Despite this, melanoma remains very challenging to treat and more knowledge on the metastatic process used by these tumours is needed. The metastasis of this aggressive tumour has been studied extensively and there is a growing literature suggesting that inflammation plays a role in many cancers [11, 12]. This study follows on from our earlier work suggesting a stimulatory effect of inflammation in melanoma [13] and is based on the clinical phenomenon of “local recurrence” of melanoma after surgical excision of the primary melanoma tumour. For some patients melanomas can re-occur in the excised wound bed some months after excision of the primary tumour sites. One theory which has been investigated to a slight extent is that the act of primary melanoma excision creates a wound bed environment with upregulation of degradative enzymes and pro-inflammatory cytokines which is conducive to the subsequent attachment and migration of circulating melanoma cells. This has been tested in an animal study [14] where a wound bed was created anatomically distant to the site of primary melanoma. Post-excision of the primary melanoma “local recurrence” occurred at this wound bed site. This argues strongly in support of the hypothesis that the factors which are part of the physiological response to wounding are also unfortunately conducive to melanoma attachment, migration and invasion.

In normal wound healing the sequence of events which occurs is complex and it is very difficult to study the effects of mechanical trauma separately to the effects of pro-inflammatory cytokines. Also it is not possible to ask questions of whether the stromal cells on their own stimulate or inhibit melanoma invasion or whether it is a combination of the keratinocytes and the fibroblasts which influence tumour progression. Tissue engineered models of skin offer opportunities to look at some of these factors in isolation and our experiences to date have yielded some interesting findings which could not readily have been detected from conventional 2D cell culture experiments or indeed from animal models. Essentially, our *in vitro* results show melanoma cell invasion influences and is influenced by adjacent skin cells [15, 16] and our evidence based on both 2D and 3D models suggests that inflammation can exacerbate melanoma invasion [15, 16] and anti-inflammatories can reduce invasion [16–19].

The aim of this study therefore was to develop *in vitro* models of wounded and inflamed skin to learn more about the factors that may influence melanoma invasion and hence develop new approaches to reducing this. We developed an incisional human skin wound model and looked at the ability of three different melanoma cells varying in their metastatic potential to invade in this model under non-wounded and wounded conditions and we looked at the impact of adding a major pro-inflammatory cytokine TNF-α (Tumour Necrosis Factor-alpha), and fibrin (which would normally be found in a wound healing response) to these wounds. Finally, we looked at the effect of adding a non-steroidal anti-inflammatory, ibuprofen, to melanoma cells invading in this model.

These results provide further evidence for a wound healing environment being conducive to melanoma invasion but encouragingly provide evidence that the local addition of a clinically acceptable anti-inflammatory such as ibuprofen can attenuate this invasion.

## Materials and Methods

### Materials

Cell culture media, antibiotics and additives were purchased from Gibco BRL (Life Technologies, Paisley, UK). Foetal calf serum (FCS), Pluronic F-127 polymer. Fibrinogen, thrombin (from bovine plasma) and sodium hydrogen carbonate were all purchased from Sigma-Aldrich (Poole, Dorset, UK). TNF-α was purchased from GlobePharm Limited (Esher, UK); Trypsin/ethylenediamine-tetraacetic acid (EDTA) and phosphate-buffered saline (PBS) tablets were purchased from Oxoid Ltd. (Basingstoke, UK). Paraformaldehyde was purchased from BDH/Merck, Poole, Dorset, UK. Rabbit-derived S-100 antibody; mouse-derived HMB45 antibody; and, mouse anti-cytokeratin AE1AE3 antibody were purchased from Dako (Carpintera, CA, USA).

### Culture of Melanoma Cells

The HBL cell line was derived from a lymph node metastasis of a nodular melanoma established in one of our laboratories [20]. The A375-SM cell line was a generous gift from Professor IJ Fidler (USA) via Professor MJ Humphries (University of Manchester, UK). A375 was established in culture from a lymph node metastasis of a 54-year-old female [21]. These cells are heterogeneous in nature and a highly metastatic variant (A375-SM) was established in culture from lung metastases produced by parental A375 cells growing subcutaneously in nude mice [22]. The C8161 melanoma line was kindly donated by Professor F Meyskens (University of California, Irvine, USA) via Professor M Edwards (University of Glasgow, UK). C8161 was established from an abdominal wall metastasis [23].

C8161 and A375SM melanoma cells were cultured in Eagle`s modified essential medium (EMEM) supplemented with 10% FCS, 2mM L-Glutamine, 100 units/ml penicillin, and 100μg/ml streptomycin sulphate, 1.2 mg/ml amphotericin B, 1.5% (of 100x stock solution) vitamin concentrate, 1mM sodium pyruvate, 1% non-essential amino acids (NEA) and 0.187% sodium hydrogen carbonate. Cells were incubated at 37°C in a humidified 5% carbon dioxide/95% air environment under standard conditions and passaged prior to confluence using 0.002% ethylenediamine tetraacetic acid (EDTA). Cells were used between passages 30–60 for experimentation.

HBL cells were maintained in melanoma culture medium, which consists of Ham’s F10 medium supplemented with 5% FCS; 5% NBCS; 2 x 10^−3^ mol/L glutamine; 100 IU/mL penicillin and 100 μg/mL streptomycin. Cells were passaged when 80–90% confluent.

### Production of de-epidermised acellular dermis (DED)

Skin was obtained from split-thickness skin grafts (STSGs) from patients undergoing routine plastic surgery for breast reduction and abdominoplasties. Patients gave written informed consent for tissue not required for their treatment to be used for research purposes. All tissue was banked on an anonymous basis under a Human Tissue Authority Research Tissue Bank Licence, Number 12179. Ethical approval for this Research Tissue Bank was obtained from the National Research Ethics Service (NRES) Committee Yorkshire & The Humber–Sheffield (REC ref: 15/YH/0177).

STSGs were stored in PBS for a minimum of 48 h and then immersed in sterile 1 mol/L sodium chloride for 18h, resulting in an acellular de-epidermised human dermis (DED). The epidermis was separated from the underlying dermis using forceps and the remaining DED samples were washed several times in PBS, then stored in DMEM supplemented with 5% FCS, 2 x 10^−3^ mol/L glutamine, 100 IU/mL penicillin, 100 μg/mL streptomycin and 0.625 μg/mL amphotericin B. This medium is referred as to fibroblast culture medium (FCM).

### Culture of Keratinocytes

STSGs were obtained as previously described. Samples of this skin were cut into 0.5 cm^2^ pieces using a scalpel blade and were incubated overnight (12–24 h) at 4°C in 0.1% w/v trypsin. FCS was added to neutralize the trypsin and the epidermal and dermal layers were carefully separated using a pair of forceps with fine points. A scalpel blade was used to gently scrape basal keratinocytes from the under surface of the epidermis and the papillary surface of the dermis.

The cells were collected into a mixture of FCS and PBS in sterile 25 ml universal containers. The cell suspension was then centrifuged at 300g for 5 min and the cells were resuspended in a known volume of keratinocyte culture medium (KCM). This medium consists of DMEM and Ham’s F12 medium in a 3:1 ratio supplemented with 10% FCS, 10 ng/mL EGF; 0.4 μg/mL hydrocortisone; 1.8 x 10^−4^ mol/L adenine; 5 μg/mL insulin; 5 μg/mL transferrin; 2 x 10^−3^ mol/L glutamine; 2 x 10^−7^ mol/L tri-iodo thyronine; 0.625 μg/mL amphotericin B; 100 IU/mL penicillin and 100 μg/mL streptomycin. A cell count was carried out using a haemocytometer, and trypan blue exclusion was used to assess cell viability. Keratinocytes were not used above passage 3.

### Culture of Fibroblasts

STSGs were trypsinized as described previously for the isolation of keratinocytes. The epidermal and dermal layers were separated and the dermal pieces were washed several times in sterile PBS and then finely minced with a scalpel blade. The dermal mince was incubated at 37°C overnight in 10 ml of a 0.5% collagenese A solution. The following day, the collagenase digest was spun down in a centrifuge at 300 g for 10 min, the supernatant was discarded and the pellet of cells was resuspended in FCM. Cells were passaged when fibroblasts reached 80–90% confluence using 2 ml of a 1:1 mixture of 0.1% w/v trypsin and 0.02 5 w/v EDTA per flask. Fibroblasts were used between passages 4 and 8.

### Tissue Engineered (TE) Human Skin and Melanoma Models

De-cellularised human skin was prepared as described above which resulted in a DED, with retained basement membrane antigens. Dermal fibroblasts were seeded onto the reticular surface of DED at a concentration of 1 x 10^5^ cells/construct ring. After 48 h, melanoma cells (5 x 10^4^ cells/construct ring) and epidermal keratinocytes (1 x 10^6^ cells/construct ring) were added to the papillary DED surface. After 48 h, the tissue engineered skin constructs were raised to an air-liquid interface. Non-wounded constructs were cultured for 15 days and the culture medium was changed every 2–3 days. Paraffin-fixed histology sections (4 μm) were made for H&E (Haematoxylin and Eosin) staining. For a full characterisation of these models see [15].

After 2–3 days at an air-liquid interface, the skin constructs were ready for wounding. The wounds were made using a scalpel blade, making a simple cut in a cell seeded area through the epidermal layer into the dermis. In some experiments 30–50μl fibrin clot solution plus 300U/ml TNF-α were also added to the wounds.

Once the TE skin models had been exposed to an air-liquid interface for 10 days, they were wounded and cultured for another 9 days–so total elapsed time was 19 days. They were fixed in 10% phosphate buffered formaldehyde for at least 24h before routine histological staining for H&E. All samples were assessed for overall morphology, examining the presence and organisation of keratinocytes and fibroblasts in the skin and assessing melanoma cell invasion into the reconstructed human skin. In some experiments immunohistochemistry was undertaken to identify keratinocytes (using AE1AE3 antibody), HBL cells (using S100) and A375SM (using HMB45 antibody). (Unfortunately C8161 cannot be identified with either S100 or HMB45—please see [15] for a full description).

To quantify melanoma cell invasion into the constructs, an invasion score was used as previously established by [15]. The score was based on the number of cells invaded into the dermis counted in each 3D skin model slide. A score of 0 indicates no cells detected in the dermis, a score of 1 represents 6–15 cells in the dermis, a score of 2 represents 21–30 cells in the dermis and finally, a score 3 or above was taken as evidence of clear invasion of cells (greater than 30 cells per section).

### Statistical Analysis

To analyse the differences between melanoma cell invasion in 3D tissue engineered skin models, a Kruskal-Wallis 1-way ANOVA test appropriate for multiple comparisons was used to compare all pairs. This analysis was undertaken using IBM SPSS (Statistical Package for the Social Sciences) Version 22.

## Results

### Invasion of melanoma cells into tissue engineered skin

**Fig 1:**
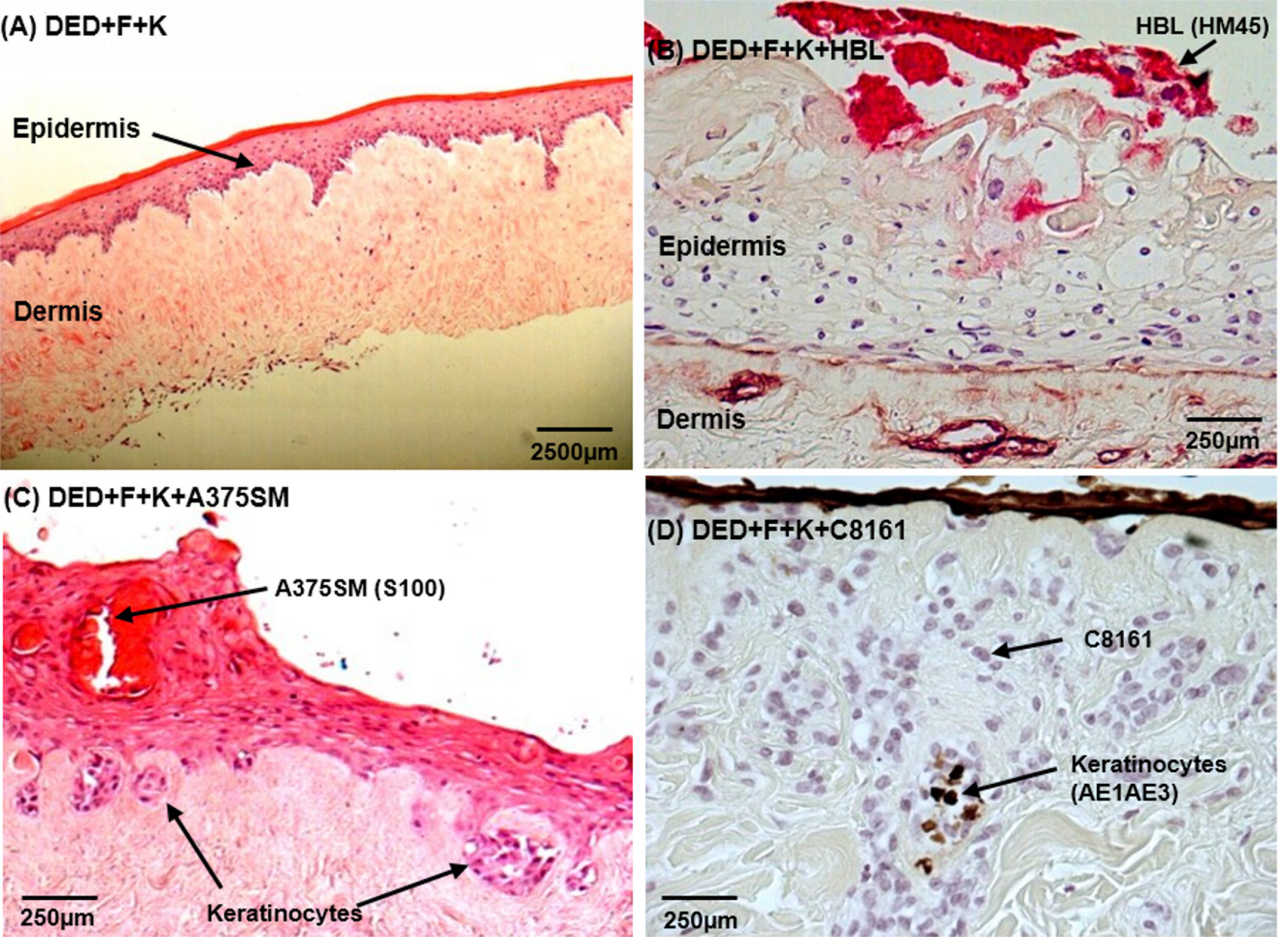
Invasion of melanoma cells into tissue engineered skin. (A) Control TE skin constructs to which fibroblasts and keratinocytes were added to the DED (4x magnification); (B) Skin cells (fibroblasts and keratinocytes) and HBL melanoma cells in DED stained with HM45 antibody staining (40x magnification); (C) Skin cells (fibroblasts and keratinocytes) and A375SM cells stained with S100 antibody staining for A375SM cells (40x magnification); (D) Skin cells (fibroblasts and keratinocytes) and C8161 cells in DED, AE1AE3 antibody staining for keratinocytes only (40x magnification). (DED = De-Epidermised acellular Dermis; F = Fibroblasts; K = Keratinocytes).

This TE skin model normally has a well defined epidermal layer securely attached to the underlying dermis with a reasonable degree of epithelial organisation in the epidermis ranging from basal cells with large nuclei to superficial layers lacking nuclei. The dermis contains a relatively low density of cells—identified as dermal fibroblasts. This is shown in Fig 1A at x4 magnification where the organisation of the epidermis and dermis is similar to that of normal human skin. We have deliberately worked with 3 melanoma cell lines selected to be very different in their invasive behaviour, HBL cells which are not normally very invasive and can be identified by staining with HMB45, A375SM cells–more aggressively invasive and these can be stained with S100 and a third cell line, C8161, which is highly invasive. These cells cannot be stained with either HMB45 or S100. Accordingly we stained the keratinocytes present in these TE constructs with an antibody to AE1AE3.

**Fig 2:**
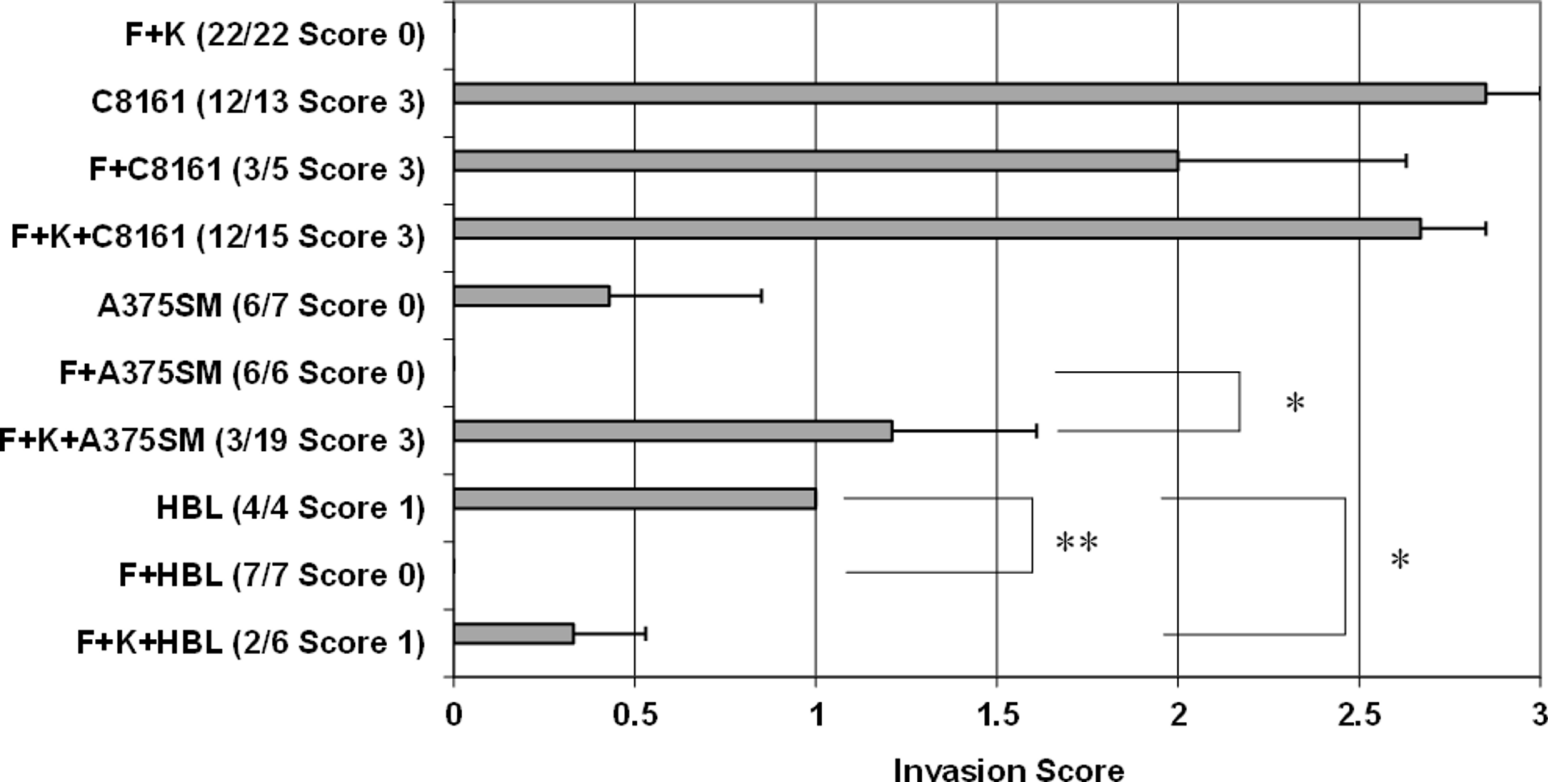
Quantitation of melanoma cell invasion into the 3D skin models. The composition of cells in these models is given followed by the number of skin constructs. The maximum invasion score is indicated in brackets. The histograms show the mean +1SEM of all scores. * indicates p<0.05; ** indicates <0.01. (F = fibroblasts, K = keratinocytes).

Fig 1B shows HBL cells did not disturb the epithelial layer to a great degree. Fig 1C shows that A375SM cells were present in the deeper layers of the epidermis. Also in these models keratinocytes are shown to invade the papillary layer of the dermis in the presence of A375SM cells. (This is aberrant behaviour for these cells as can be seen from quantitative analysis in Fig 2). In Fig 1D the behaviour of C8161 cells is shown. These cells invaded into the papillary and reticular layers of the dermis. Some keratinocytes were also present in the dermal layer in these skin models identified by staining with AE1AE3.

Table 1 compares the invasion scores for the 3 cell lines which shows that the difference in invasion between the HBL and A375SM cells was not significant in this model, whereas the invasion of C8161 cells was statistically greater than either of the other two cell types.

**Table 1:** Comparisons of invasion scores in TE models containing different melanoma cell lines.

Skin Constructs	Invasion Score	p-value
[F+K+HBL] *vs* [F+K+A375SM]	0.3 *vs* 1.21	0.172
[F+K+HBL] *vs* [F+K+C8161]	0.3 *vs* 2.67	0.000***
[F+K+A375SM] *vs* [F+K+C8161]	1.21 *vs* 2.67	0.001**

F = Fibroblasts; K = Keratinocytes

**p<0.01

***p<0.001.

### Influence of skin cells on melanoma invasion

In these experiments (Fig 2) TE constructs were prepared so that they contained fibroblasts and keratinocytes (as in normal human skin), melanoma cells alone, melanoma cells and fibroblasts or melanoma cells and fibroblasts and keratinocytes. The extent of cellular invasion into the dermis was then quantified. As can be seen in the standard TE constructs there was no evidence of epidermal cell invasion into the dermis in 22 out of 22 constructs. This result was expected as in these TE models there were no melanoma cells present and hence, no influence of melanoma cells on normal skin cell organisation in these 3D skin models. For HBL cells, these were moderately invasive into the dermis on their own but the presence of fibroblasts completely blocked their invasion (p = 0.001) while the presence of keratinocytes and fibroblasts slightly reduced their invasion (p = 0.036). On further examination HBL cells in constructs containing fibroblasts and keratinocytes were slightly (not significantly) more invasive compared to HBL in skin constructs containing only fibroblasts. A375SM cells were not very invasive on their own in the dermis (invasion score of 0.5 out of 3.0) but there was no invasion of these cells at all in the presence of fibroblasts. A375SM cells in constructs containing fibroblasts and keratinocytes were significantly more invasive than A375SM cells in the presence of fibroblasts (p = 0.011). Comparing the melanoma invasion seen in the presence of both keratinocytes and fibroblasts to that seen in the presence of fibroblasts alone it is clear that fibroblasts on their own reduced HBL and A375SM invasion whereas keratinocytes tended to exacerbate it.

In contrast, C8161 cells were extremely invasive (achieving a near maximal score of 3.0 out of 3.0 for 12 out of the 13 TE constructs examined). The addition of fibroblasts had no significant effect on this, nor did the addition of fibroblasts and keratinocytes. This melanoma cell line remained aggressively invasive in all experiments and was always more invasive than A375SM and HBL cells under the same conditions.

### Development of wounded TE skin model with inflammation

**Fig 3:**
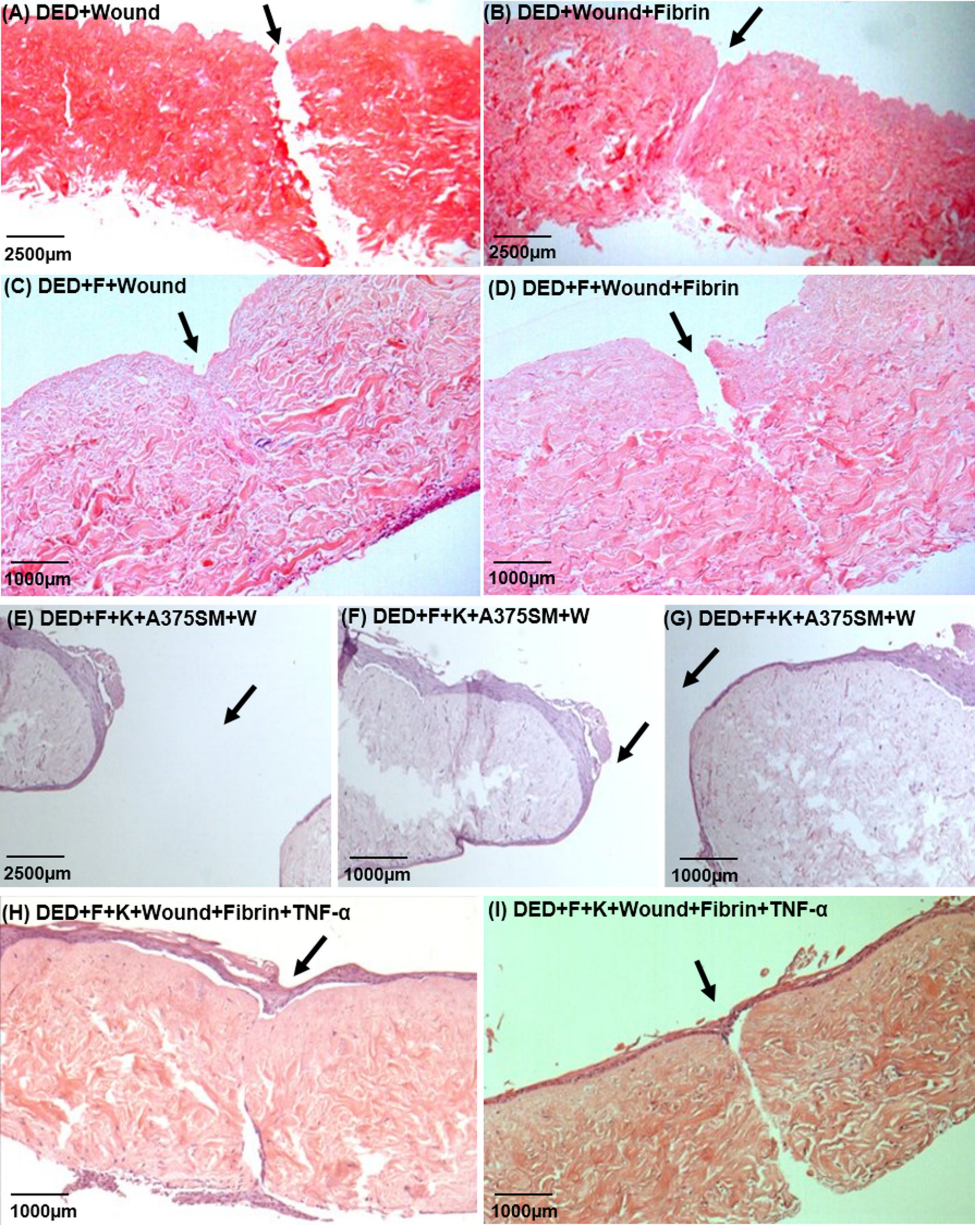
Effect of mechanical wounding and addition of fibrin and TNF-α on wound healing in TE skin. (A) DED+Wound (4x magnification); (B) DED+Wound+Fibrin (4x magnification); (C) DED+Fibroblasts+Wound (10x magnification); (D) DED+Fibroblasts+Wound+Fibrin (10x magnification); Wounded standard skin constructs (E, F and G) (4x, 10x and 10x magnifications respectively). Wounded standard skin constructs with the addition of fibrin and TNF-α (H and I) (10x magnification). The site of the wound is indicated by an arrow. DED = De-Epidermised acellular Dermis, F = Fibroblasts, W = Wound.

Fig 3 shows the development of the wounded skin model. A simple incisional wound was made in the acellular DED, as shown in Fig 3A. This cannot heal as it lacks cells. Fig 3B shows acellular DED to which fibrin has been added following wounding. The presence of fibrin physically reconnected both sides of wound. Fig 3C shows DED plus fibroblasts plus wounding. This wound completely healed by 14 days due to the presence of the fibroblasts. Fig 3D shows a wounded DED to which was added fibroblasts and a fibrin clot and this took longer to achieve to complete healing. Fig 3E shows TE skin constructs containing keratinocytes, fibroblasts and one of the melanoma cell lines, A375SM, post-wounding (at 4x magnification). This shows a wide injury that actually increased in size over 14 days. A higher magnification can be seen in Fig 3F and 3G. This open wound in a TE skin containing A375SM cells may indicate a possible delay in healing caused by the presence of melanoma cells. Both Fig 3H and 3I show TE skin containing keratinocytes and fibroblasts post-wounding and the addition of fibrin and TNF-α. In Fig 3H there is damage to the dermis but the wound has largely healed and there is a continuous epidermis. In Fig 3I the epidermal layer is continuous but the underlying dermal wound is still evident. Comparing these two skin constructs, the results suggest that epidermal healing may be independent of dermal healing in this model.

Table 2 summarises the various conditions that were evaluated in the development of a wounded skin model containing the pro-inflammatory cytokine TNF-α. From this, it appears that a wounded skin model containing fibroblasts or fibroblasts plus fibrin has a good chance of healing. A wounded skin model containing keratinocytes and fibroblasts (as shown in the examples of Fig 3H and 3I) may show partial healing, particularly of the epidermal layer.

**Table 2:** Effect of fibrin clot, TNF-α and skin cells on wound healing.

Fibroblasts	Wound	Fibrin	TNF-α	Keratinocytes	Result
-	-	-	-	-	Normal DED
-	-	+	-	-	Normal DED
-	+	-	-	-	Open wide wound (Fig 3A)
-	+	+	-	-	Narrowed wound (Fig 3B)
+	-	-	-	-	Normal Dermis + F
+	-	+	-	-	Normal Dermis + F
+	+	-	-	-	Healed (Fig 3C)
+	+	+	-	-	Narrowed wound (Fig 3D)
+	+	-	-	+	Open wide wound (Fig 3E, 3F and 3G)
+	+	+	+	+	Epidermis healed, healed wound (Fig 3H)
+	+	+	+	+	Epidermis healed, narrowed wound (Fig 3I)

Fibrin = Fibrin Clots, TNF = 300U/ml TNF-α, F = Fibroblasts.

### Effect of sodium ibuprofen on melanoma invasion

**Fig 4:**
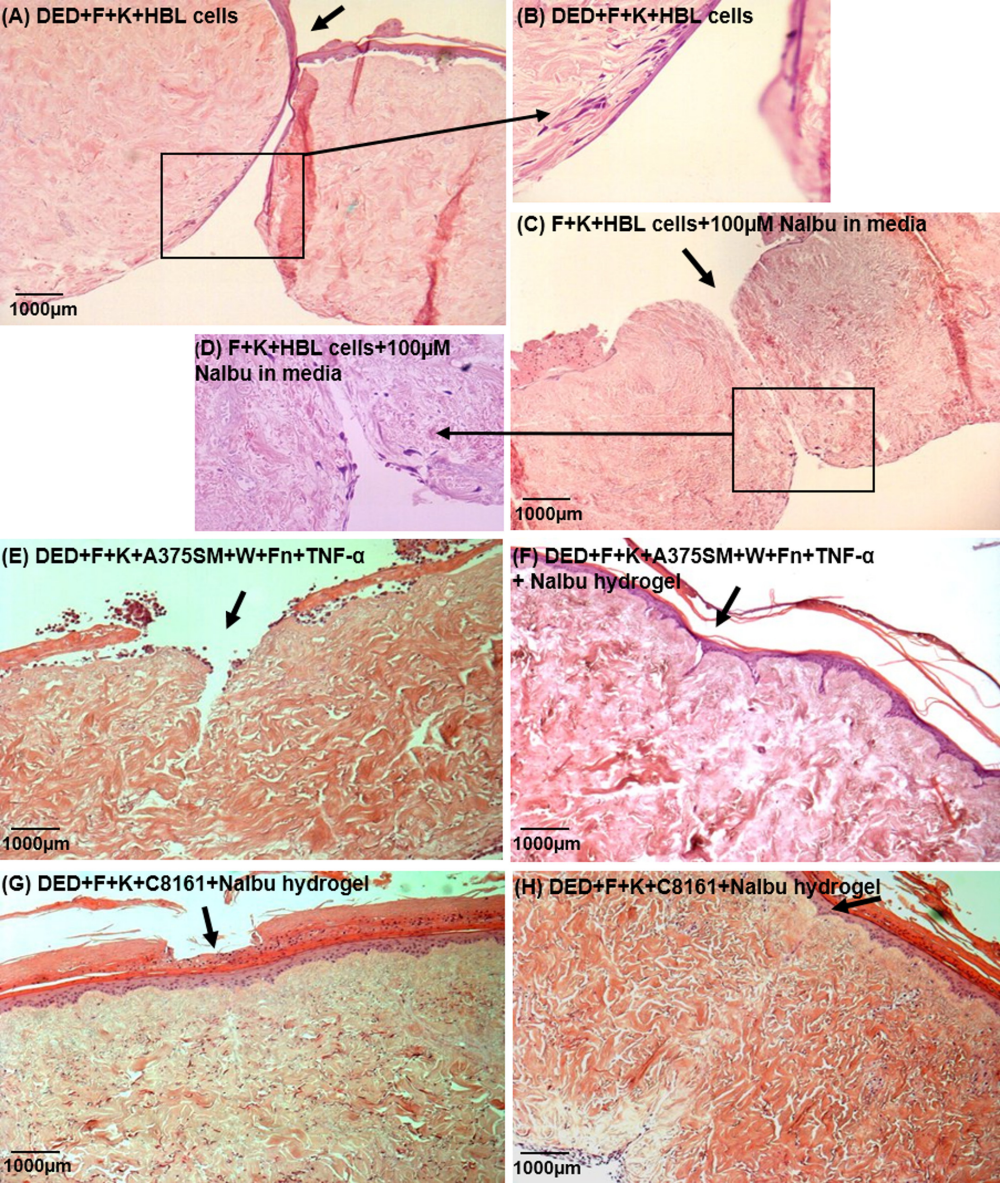
Examples of the effect of sodium ibuprofen on melanoma invasion. **Wounded skin constructs.** (A and B) DED+F+K+HBL cells (10x and 40x magnifications respectively); (C and D) F+K+HBL cells+100μM NaIbu in media (10x and 40x magnifications respectively); (E) constructs containing DED+F+K+A375SM that have been wounded and a fibrin clot placed in the wound and which have also received TNF- α (300U/ml) (10x magnification); (F) A375SM Melanoma skin construct to which were added fibrin clots plus TNF-α (300U/ml) and a further treatment with 100μM NaIbu hydrogel (30μl volume), (10x magnification); (G) Effects of NaIbu hydrogel on wound healing of wounded melanoma skin constructs, DED+F+K+C8161; (H) F+K+C8161+NaIbu hydrogel 131μM (10x magnification). The site of the wound is indicated by an arrow. (DED = De-Epidermised acellular Dermis, F = Fibroblasts, K = Keratinocytes, W = Wound).

Fig 4 shows examples of melanoma cells invading into deliberately wounded skin and the impact of adding ibuprofen. Fig 4A and 4B shows that HBL cells appear to be in the dermis following wounding. Fig 4C and 4D indicate that following the addition of sodium ibuprofen there may be less invasion.

With respect to A375SM cells these were moderately invasive and more invasive following wounding (see almost healed wound) (Fig 4E). Fig 4F shows that following the addition of sodium ibuprofen gel there was reasonable healing and less invasion.

With respect to C8161 cells their invasion post wounding is shown in Fig 4G. The most striking finding was that the dermis appeared to be very compact and intact in the presence of these cells even following wounding. Detailed examination showed extensive invasion of these C8161 cells throughout the dermis despite the compact integrity of the dermis. These cells were identified as melanoma on the basis of their large nuclei and the fact that they did not stain for keratinocytes and were far too plentiful for fibroblasts (which also had smaller nuclei). This invasion was not affected by the addition of ibuprofen as shown in Fig 4H where dermal integrity remained very good but C8161 penetration was extensive throughout the dermis.

**Fig 5:**
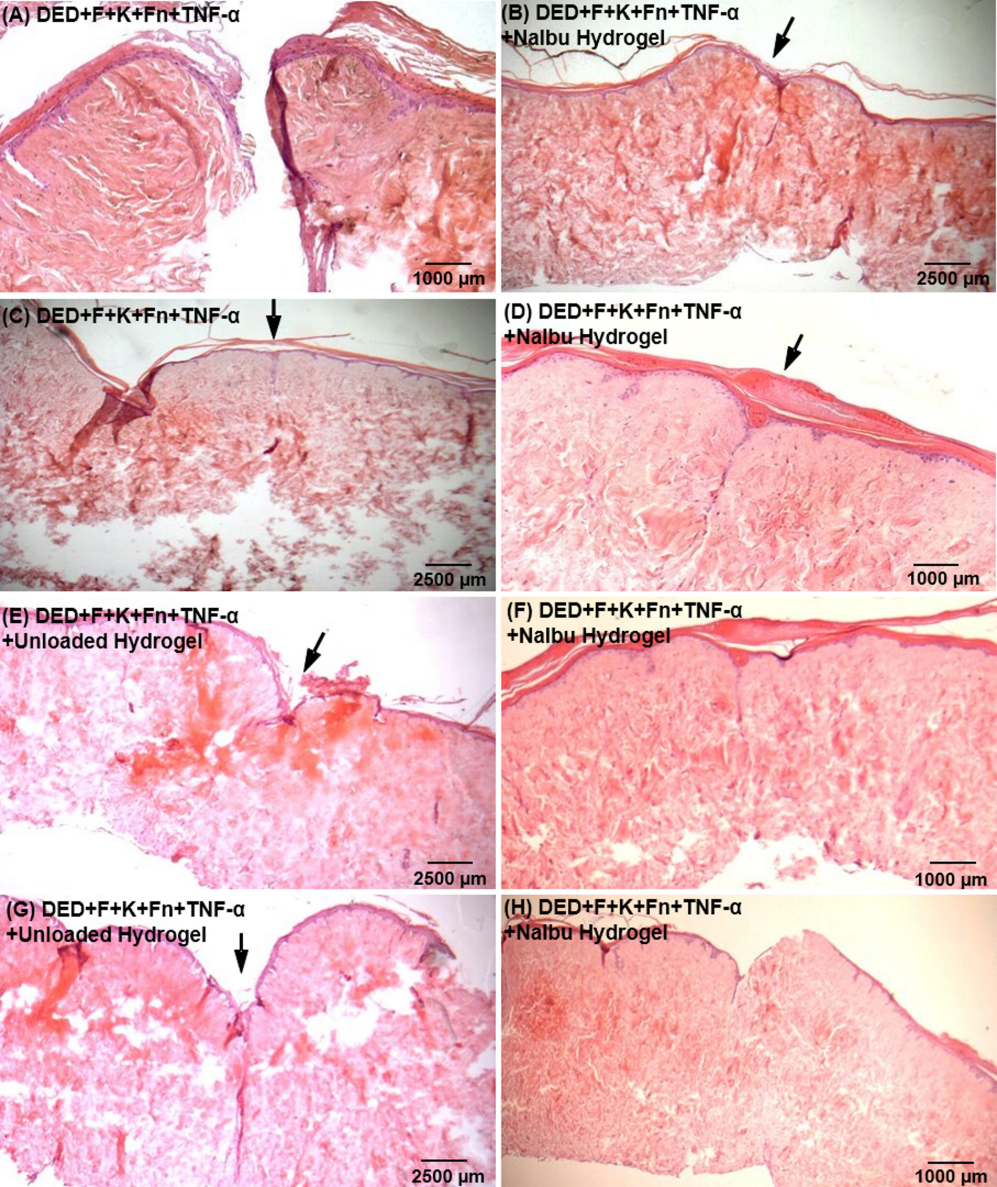
Effects of Ibuprofen on wounded melanoma skin construct model. All constructs contained fibroblasts, keratinocytes and A375SM cells and fibrin clots plus 300 U/ml TNF-α (as in A and C). To (E) and (G) was added unloaded gel. To (B), (D), (F) and (H) were added 100μM NaIbu hydrogel. (B), (C), (D), (E), (G) and (H) = 4x magnification; (A) and (F) = 10x magnification. The site of the wound is indicated by an arrow. DED=De-Epidermised acellular Dermis, F=Fibroblasts, K=Keratinocytes, W=Wound.

Examples of the impact of sodium ibuprofen on melanoma invasion are shown in Fig 5 which shows results from four such experiments where it was noticeable that in these models, which were all deliberately wounded and then a fibrin clot and 300U/ml TNF-α added, the integrity of the resulting dermis was very poor and the wound repair was variable in the absence of ibuprofen (see Fig 5A, 5C, 5E and 5G). In contrast, the wound repair and the quality of the dermis were much better where ibuprofen was present (see Fig 5B, 5D, 5F and 5H).

The effect of the presence of melanoma cells on dermal integrity was then analysed asking to what extent did melanoma cells damage or improve the dermal integrity.

Using a simple description of dermal integrity as either good or poor quality this analysis was as follows. In standard TE skin constructs 20 out of 22 (91%) of experiments were judged to have good quality dermis (as seen in Fig 1A). In Fig 4 experiments containing HBL cells, 4 had good quality dermis (66%) and 2 poor quality dermis. In 19 experiments containing A375SM cells, 15 (79%) showed a good dermal quality while 4 (21%) had a poor dermal quality. Interestingly all 15 skin constructs containing C8161 cells showed good dermal quality (100%). Thus there is some suggestion that HBL and A375SM cells reduced dermal quality but clearly C8161 did not.

### Quantitation of the effects of wounding and inflammation on melanoma cell invasion in TE skin

**Fig 6:**
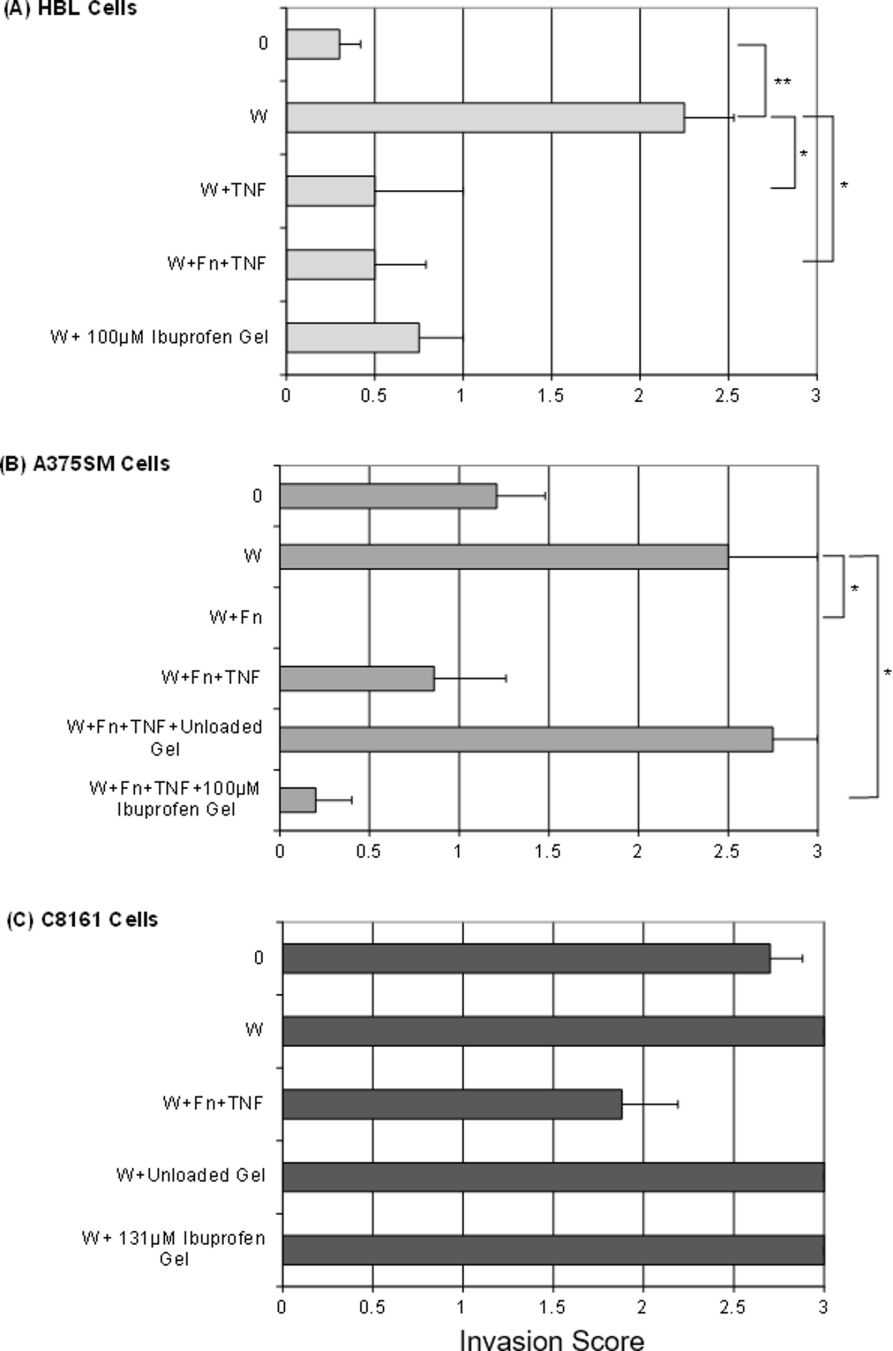
Quantitation of the effects of wounding and Ibuprofen on melanoma cell invasion in TE skin constructs. (A) with HBL cells, (B) with A375SM cells and (C) with C8161 cells. To the constructs were added W=Wound, Fn=Fibrin clots, TNF=300U/ml TNF-α, NaIbu=Ibuprofen sodium salt as shown. Values (bars) are expressed as Mean+SEM of invasion score. * indicates p<0.05; ** indicates p<0.01.

Fig 6 shows the summarised results for a large number of experiments looking at the impact of wounding and the pro-inflammatory cytokine TNF-α and the anti-inflammatory ibuprofen on melanoma invasion into TE skin. This data is also summarised in Table 3 which indicates the number of experiments undertaken and the individual experimental results. In Fig 6A, HBL cells are weakly invasive but there is a significant increase in invasion in the dermis following mechanical wounding (p˂0.01). Addition of TNF-α in media or fibrin clots and TNF-α or sodium ibuprofen did not reduce invasion of HBL cells (p>0.05). Invasion of HBL in wounded skin constructs, however, was reduced by the addition of TNF-α (p=0.036) or fibrin plus TNF-α (p=0.008). The addition of ibuprofen on its own did not significantly reduce the invasion of these cells.

**Table 3:** Effects of NSAID on melanoma invasion in wounded skin constructs.

	Invasion Score								
Skin Constructs	Total Number of Experiments	0	0.5	1	1.5	2	2.5	3	Mean Invasion Score
F+K	22	22							0
F+K+W	1	1							0
F+K+W+Fn+TNF-α ^#^	3	2					1		0.83
F+K+HBL	6	4		2					0.3
F+K+HBL+W	6				2	1	1	2	2.25 **
F+K+HBL+W+TNF-α (in media)	2	1		1					0.5
F+K+HBL+W+Fn+TNF-α	6	3		3					0.5
F+K+HBL+W+ NaIbu 100μM	2		1	1					0.75
F+K+A375SM	19	7	2	1	2	2	2	3	1.21
F+K+A375SM+W	2					1		1	2.5
F+K+A375SM+W+Fn	3	3							0
F+K+A375SM+W+Fn+TNF-α	7	3		3				1	0.86
F+K+A375SM+W+Fn+TNF-α+Unloaded hydrogel	2						1	1	2.75
F+K+A375SM+W+Fn+TNF-α+NaIbu 100μM hydrogel	5	4		1					0.2
F+K+C8161	15			2		1		12	2.67
F+K+C8161+W	1							1	3
F+K+C8161+W+Fn+TNF-α	4			1		2	1		1.88
F+K+C8161+W+Unloaded hydrogel	2							2	3
F+K+C8161+W+NaIbu 131μM hydrogel	2							2	3

^#^ = Invasion of Keratinocytes

F = Fibroblasts, K = Keratinocytes, W = Wound, Fn = Fibrin clots, TNF = 300U/ml TNF-α, Unloaded Gel = Hydrogel without Sodium Ibuprofen. NaIbu100μM Gel = Sodium Ibuprofen hydrogel 100μM.

** p˂0.01.

In Fig 6B results with A375SM cells are seen. These are moderately invasive cells. Invasion was slightly increased following wounding (although not significantly so, p = 0.166). The addition of fibrin reduced invasion but not to a significant level (p = 0.071). The addition of TNF-α did not affect invasion (p = 0.833). The addition of a control hydrogel did not reduce invasion (p = 0.114) but when this hydrogel contained ibuprofen cell invasion was decreased although not to a significant level (p = 0.08). Compared to wounded A375SM TE models, invasion was significantly reduced by the addition of fibrin (p = 0.018) and by Ibuprofen hydrogel (p = 0.022). A significant reduction was observed in A375SM TE models treated with sodium ibuprofen hydrogel compared to the ones treated with a control hydrogel (p = 0.014).

In Fig 6C C8161 cells showed extensive invasion pre-wounding and post-wounding and this was not changed by the subsequent addition of fibrin plus TNF-α or by the addition of ibuprofen (p = 0.064). Essentially, these cells remained aggressively invasive under all conditions.

In summary, the three melanoma cell lines showed similar invasion scores in wounded skin constructs. Ibuprofen hydrogel reduced melanoma cell invasion in two of the least invasive cell lines. A summary of these results can be seen in Table 4.

**Table 4:** Comparisons of invasion scores in wounded melanoma TE models.

Skin Constructs	Invasion Score	p-value
[F+K+HBL] *vs* [F+K+HBL+W]	0.3 *vs* 2.25	0.001**
[F+K+HBL] *vs* [F+K+HBL+W+TNF-α (media)]	0.3 *vs* 0.5	0.788
[F+K+HBL] *vs* [F+K+HBL+W+Fn+TNF-α]	0.3 *vs* 0.5	0.734
[F+K+HBL] *vs* [F+K+HBL+W+NaIbu100μM]	0.3 *vs* 0.75	0.485
[F+K+HBL+W] *vs* [F+K+HBL+W+TNF-α (media)]	2.25 *vs* 0.5	0.036*
[F+K+HBL+W] *vs* [F+K+HBL+W+Fn+TNF-α]	2.25 *vs* 0.5	0.008**
[F+K+HBL+W] *vs* [F+K+HBL+W+NaIbu100μM Gel]	2.25 *vs* 0.75	0.096
[F+K+A375SM] *vs* [F+K+A375SM+W]	1.21 *vs* 2.5	0.116
[F+K+A375SM] *vs* [F+K+A375SM+W+Fn]	1.21 *vs* 0	0.071
[F+K+A375SM] *vs* [F+K+A375SM+W+Fn+TNF-α]	1.21 *vs* 0.86	0.833
[F+K+A375SM] *vs* [F+K+A375SM+W+Fn+TNF-α+Unloaded Gel]	1.21 *vs* 2.75	0.114
[F+K+A375SM] *vs* [F+K+A375SM+W+Fn+TNF-α+ NaIbu100μM Gel]	1.21 *vs* 0.2	0.08
[F+K+A375SM+W] *vs* [F+K+A375SM+W+Fn]	2.5 *vs* 0	0.018*
[F+K+A375SM+W] *vs* [F+K+A375SM+W+Fn+TNF-α]	2.5 *vs* 0.86	0.167
[F+K+A375SM+W] *vs* [F+K+A375SM+W+Fn+TNF-α+Unloaded Gel]	2.5 *vs* 2.75	0.885
[F+K+A375SM+W] *vs* [F+K+A375SM+W+Fn+TNF-α+ NaIbu100μM Gel]	2.5 *vs* 0.2	0.022*
[F+K+A375SM+W+Fn+TNF-α+Unloaded Gel] *vs* [F+K+A375SM+W+Fn+TNF-α+ NaIbu100μM Gel]	2.75 *vs* 0.2	0.014*
[F+K+HBL+W] *vs* [F+K+A375SM+W]	2.25 vs 2.5	0.557
[F+K+HBL+W] *vs* [F+K+C8161+W]	2.25 *vs* 3	0.557
[F+K+A375SM+W] *vs* [F+K+C8161+W]	2.5 *vs* 3	0.557

F = Fibroblasts, K = Keratinocytes, W = Wound, Fn = Fibrin clots, TNF = 300U/ml TNF-α, Unloaded Gel = Hydrogel without Sodium Ibuprofen. NaIbu100μM Gel = Sodium Ibuprofen hydrogel 100μM.

* p˂0.05

** p˂0.01.

### Effects of Melanoma Cells on Wound Healing

Finally, the impact of the melanoma cells on wound healing over 15 days was examined. Healing was assessed as no healing, intermediate healing or good healing. This was a subjective and observational method considering an open wound, a partially open wound (or partially healed wound) and a wound completely closed from the epidermis to the dermis as summarised in Table 5. HBL containing TE skin constructs proved to be the most difficult to heal as 10 out of 14 (71.4%) did not heal. A few of these skin constructs (3 out of 14, or 21.4%) showed some intermediate healing and only 1 out of 14 (10%) showed good healing. A375SM cells were slightly better at healing as 6 out of 14 (43.8%) healed, with an intermediate healing in 5 out of 14 (35.7%) whilst only 3 out of 14 (21.4%) did not heal. For C8161 cells 7 out of 7 wounded TE constructs healed completely (100%).

**Table 5:** Effects of Melanoma Cells on Wound Healing in TE Skin.

Skin Constructs			
	HBL Cells	A375SM Cells	C8161 Cells
No Healing	15	3	0
Intermediate Healing	3	6	0
Good Healing	2	9	7

This is an unexpected result as one might expect wound healing to relate simply to the exent of melanoma invasion into the dermis. Thus one would expect that the least invasive melanoma cells (HBL) would not affect wound healing whilst the most invasive melanoma cells (C8161) would cause more tissue destruction with delayed wound healing. The only results which were as expected were shown by A375SM cells which were moderately invasive and led to TE constructs with moderate wound healing characteristics.

## Discussion

The aim of this study was to design physiologically relevant 3D models in which to investigate aspects of wound healing, inflammation and the use of a common anti-inflammatory on melanoma invasion.

The TE constructs were prepared so that they contained all combinations of cells—a great advantage of using these 3D models as clearly one cannot do such experiments *in vivo*. Each of the melanoma cells we investigated behaved very differently (as expected from our selection of melanoma cell lines of varying metastatic potential). Indeed each melanoma cell had a distinctive invasive behaviour. The least invasive, HBL cells, are based on melanoma cells derived from a lymph node tumour. A375SM showed an intermediate invasion and these cells were obtained from a lymph node metastasis from parental A375 cells which were established in culture from lung metastasis grown subcutaneously in nude mice. The highest invasive potential was shown by C8161 cells which are derived from an abdominal wall metastasis. These results clearly showed three invasive behaviours of these melanoma cells in TE skin as previously reported from our laboratory by Eves *et al*. [15].

The addition of HBL cells slightly affected the normally well organised epithelium of this TE skin model [24] but there was very little disruption to the dermal layer. Addition of A375SM cells showed melanoma cells (identified by antibody staining for S100 cell marker) mainly within the epidermal layer which became poorly organised. There were also cells within the dermis which were identified as keratinocytes (by staining for AE1AE3 cell marker). These are not normally present in the dermis in the absence of melanoma cells. In contrast, the addition of C8161 cells led to an extensive florid invasion of these cells into the dermis. Very few of these cells were identified as keratinocytes by specific staining for the latter cells in the dermis. The residual epidermis was poorly organised while paradoxically the dermis itself remained compact.

HBL cells were slightly invasive into the dermis on their own but the presence of fibroblasts completely blocked their invasion. The presence of keratinocytes and fibroblasts only slightly reduced their invasion. A375SM cells were not very invasive on their own in the dermis and as for HBL cells, invasion was completely blocked by the addition of fibroblasts. However, these cells were most invasive in the presence of both keratinocytes and fibroblasts. For these two cell lines it is clear that fibroblasts alone reduced the invasion of both HBL and A375SM cells whereas keratinocytes tended to assist their invasion. In contrast, C8161 cells were extremely invasive in all experiments and unaffected by the presence of fibroblasts or keratinocytes.

We suggest the interplay beween melanoma cells and normal skin cells creates a tumour microenvironment around tumour cells.

Tumour microenvironments have been a long-term subject of study [25]. Melanoma cells may interact with other cells such as fibroblasts, endothelial cells and immune cells through paracrine and endocrine mechanisms as well as through direct contact via cell-to-cell and cell-to-matrix adhesion and gap junctional intercellular communication. In normal skin, keratinocytes tightly control melanocyte behaviour and prevent their movement into the dermis but once melanocytes become melanoma cells they acquire the ability to migrate and invade into the dermis.

In contrast to the results of this study fibroblasts have been reported to promote tumour cell invasion and are described as Carcinoma-Associated Fibroblasts (CAFs) which contribute to tumour invasion and metastasis [26]. Indeed strategic therapeutic targeting of CAFs to treat skin cancer and melanoma has been proposed [27, 28]. Fibroblasts have also been reported to decrease the cytotoxic effects of drugs on melanoma cells [29], assisting the development of chemoresistance to doxorubicin [30] in melanoma cells.

Thus the ability of fibroblasts to reduce melanoma cell invasion in our TE skin opposes most results in the literature. However there are other studies which suggest fibroblasts may oppose progression of skin cancers. One study demonstrated a decreased proportion of senescent fibroblasts in geriatric skin that may play a role in preventing the initiation of skin cancer [31]. It has been suggested that senescent fibroblasts may create a prooncogenic skin microenvironment that cooperates with mutant melanocytes to drive melanoma initiation and progression and should, therefore, be considered as a potential future therapeutic target [32]. This suggests that fibroblasts may normally retard invasion but when they become senescent they may assist invasion. [33].

Does the active or inactive state of the fibroblast influence metastasis? In our experiments the TE skin model is only cultured for around two weeks—in some of these studies we wound the TE skin. It is likely that the fibroblasts are metabolically active in these models. This suggests an area for investigation—to deliberately introduce melanoma cells into TE skin environments with fibroblasts cultured to be either metabolically active or to be senescent to explore this further.

Very recently we have confirmed that the area surrounding the melanoma tumour cells in these models is abnormal using Raman spectroscopy [33]. The most striking feature of this study was the finding of a high level of glycogen indicative of metabolically active cells surrounding the melanoma tumour cells.

Prior work from our group has shown that for some melanoma cells to invade in a 3D reconstructed skin model the presence of fibroblasts and keratinocytes are required [34]. However, it does not follow that the melanoma cells themselves upregulate degradative enzymes as we initially thought. A subsequent study from our group showed that MMP-2 and MMP-9 were not upregulated in invading melanoma cells but in the surrounding keratinocytes and fibroblasts [13, 34]. We suggest that the skin cells in modifying and organising the dermal matrix, with upregulation of matrix metalloproteinases, create a matrix conducive to melanoma cell invasion.

Keratinocytes normally play a major role in skin healing and ECM (Extracellular Matrix) breakdown and remodelling, both in their own right and by interacting with fibroblasts. Some of these activities–breakdown of the dermis–would seem to provide an easy environment for melanoma migration. Other activities—fibroblast synthesis of new collagen and its crosslinking–would seem to be anti-invasive. Thus, it appears that melanoma cells can both influence and be influenced by the surrounding skin cells.

Normal cells need contact with the ECM which regulates their anchorage and their cell cycle and the partial loss of these requirements is a hallmark of malignant cells. There is an extensive literature describing how integrin mediated contact between malignant cells and the ECM influences their behaviour [35].

In this study, HBL cells were weakly invasive but invasion in the dermis was significantly increased following mechanical wounding. For these wounded TE models invasion was reduced by the addition of TNF-α or fibrin plus TNF-α. The addition of sodium ibuprofen slightly reduced invasion but this did not achieve statistical significance almost certainly due to the relatively low number of these experiments in this study.

A375SM cells are moderately invasive cells whose invasion was slightly increased following wounding. The addition of ibuprofen hydrogel significantly reduced A375SM cell invasion in a wounded skin construct supporting the anti-invasive effects of NSAIDs in reducing melanoma cell invasion as demonstrated in an artificial wound bed.

Finally, C8161 cells showed extensive invasion pre-wounding and post wounding. This was not changed by the addition of fibrin plus TNF-α or the addition of ibuprofen. Essentially these cells remained aggressively invasive under all conditions.

Concerning TE skin healing after wounding, the wounded model containing fibroblasts or fibroblasts plus fibrin showed good healing. This may be explained by the provision of a temporary fibrin clot scaffold for cell migration and the fibroblasts to synthesise new dermal extracellular matrix components, as demonstrated by Rizzi *et al*. (2010) [36]. Standard TE skin constructs to which fibrin and TNF-α were added healed relatively well. In terms of invasion and healing properties, HBL cells were less invasive than the other melanoma cell lines but they clearly also delayed wound healing in the TE skin constructs. A375SM cells showed an intermediate level of invasiveness and wound healing. On the other hand, C8161 cells were the most invasive of all three cell lines and unexpectedly resulted in good wound healing in all constructs.

The slow healing process in wounded TE skin constructs with two of the melanoma cells (HBL and A375SM) may be relevant to the ulceration seen with some melanomas. This can be observed in a study of Balch *et al*., 2009 [37] who reported that patients with regional metastasis also had ulceration of the primary melanoma. Possibly, some melanoma cells may retard wound healing—with associated sustained inflammation. Conversely, the most aggressive melanoma cell line, C8161, invaded without influencing wound healing. This may be related to the expression of CD44 and hyaluronan on these C8161 cells, as reported by Edward *et al*. (2005) [38] but clearly the results we report in this *in vitro* model for the first time will need further investigation beyond this study.

It has been reported that following initial surgery designed to fully excise tumours, 20–50% of patients develop local recurrences [39] and survival for these patients is reduced compared to patients who do not develop local recurrences [39, 40]. Clinical experience records the phenomena of “local recurrence” of melanomas which can occur some months after excision from primary tumour sites. One theory which has only been investigated to a slight extent is that the act of excision of primary melanoma creates a wound bed environment with upregulation of degradative enzymes and pro-inflammatory cytokines which is conducive to the subsequent attachment and migration of circulating melanoma cells. This has been investigated and confirmed in an animal model [14].

There are now many studies suggesting inflammation can exacerbate tumour metastases in humans and in animals [41–42]. Our previous studies in this area have shown that TNF-α increases melanoma cell invasion and migration *in vitro* [16, 18] and that anti-inflammatory agents such as α-MSH (alpha-Melanocyte Stimulating Hormone) can reduce melanoma cell invasion and protect cells against pro-inflammatory cytokine attack in cells with the wild-type receptor (HBL) [16]. Also, we have shown aspirin [17] can effectively inhibit TNF-α -induced upregulation of NF-kappaB and ICAM-1 expression during *in vitro* migration and invasion of human melanoma cells. More recently we demonstrated similar results for Ibuprofen [19].

Our results show that in this model ibuprofen significantly reduced melanoma invasion in one out of three melanoma cell lines and showed a tendency to reduce invasion in another. It was without effect in the third, very aggressively metastatic melanoma cell line. Clearly now that this model is established it will need extending to other melanoma cell lines to establish how widespread the actions of an anti-inflammatory might be in retarding melanoma invasion. We suggest this study significantly extends our previous studies showing melanoma cells interact with skin cells in their invasion into TE skin but also shows for the first time that fibroblasts on their own tend to inhibit the invasion of melanoma cells and that ibuprofen can reduce melanoma invasion under conditions designed to model some of the key features of removal of a primary melanoma–the creation of a wound and the presence of associated inflammation. The use of a tissue engineering approach allows one to study these as separate features—something not easily possible in *in vivo* studies. These results held true for the two least invasive melanoma cell lines but unfortunately ibuprofen was without effect on the invasion of the very aggressive C8161 cells in this model.

This study suggests that non-steroidal anti-inflammatory drugs used topically may be a useful therapeutic approach to oppose the stimulatory effects of inflammation on melanoma invasion.

With respect to the use of anti-inflammatories clinically there are a few reports using systemic COX (Cyclooxygenase) inhibitors, such as Pioglitazone and Rofecoxib in the management of melanoma [43, 44]. Also Lejeune *et al*, (2006) [45] reported a case study of a patient with a metastatic melanoma of the leg who experienced sustained regression of skin metastases upon continuous single treatment with the cyclooxygenase-2 inhibitor Rofecoxib.

### Implications of this Study

In this study we propose that the spread of melanoma can be prevented by early diagnosis of a primary cutaneous melanoma and topical treatment post-surgery with a local application of a NSAID such as ibuprofen. The justification for giving the latter is to reduce the inflammatory wound bed conditions post-surgery which we suggest are conducive to melanoma metastasis.

We suggest the beneficial effects of NSAIDs on primary melanoma may indicate its use as a co-adjuvant topical agent in chemotherapy for treating melanoma but clearly more work will be needed to establish this using this or a similar model.

Clearly these results must be interpreted with caution as this is an *in vitro* study without a functioning immune system or any vasculature. The evidence presented from this 3D reconstructed skin suggests that mechanical wounding is associated with increased invasion of melanoma cells (as demonstrated by the A375SM melanoma model) but that this can be attenuated by the addition of ibuprofen (either as free drug or released from a hydrogel). If one relates these findings to the phenomena of local recurrence of melanoma then there is an argument to be made for reducing inflammation at the site of melanoma excision following excision of a primary melanoma. This could be achieved by the addition of ibuprofen either systemically or possibly, more effectively by its release from a hydrogel preparation placed on the wound bed post-surgery. Interestingly the results also indicated that there was less damage to the dermis following wounding when ibuprofen was administered in this model. Topical treatment with ibuprofen is often used as a therapeutic approach to the reduction of wound pain in patients with chronic, exuding leg ulcers [46, 47]. We suggest this study provides further evidence to support the use of topical ibuprofen at the time of melanoma surgery.

## Conclusion

In conclusion studies using a 3D tissue engineered human skin model containing melanoma cells suggest wounding increases melanoma cell invasion in an inflamed microenvironment.

These *in vitro* results support the hypothesis that the local addition of an anti-inflammatory drug such as ibuprofen can attenuate melanoma invasion and may promote healing post surgical excision of primary tumours but further work will be required with other cell lines to establish how widespread or otherwise this effect of ibuprofen may be.
